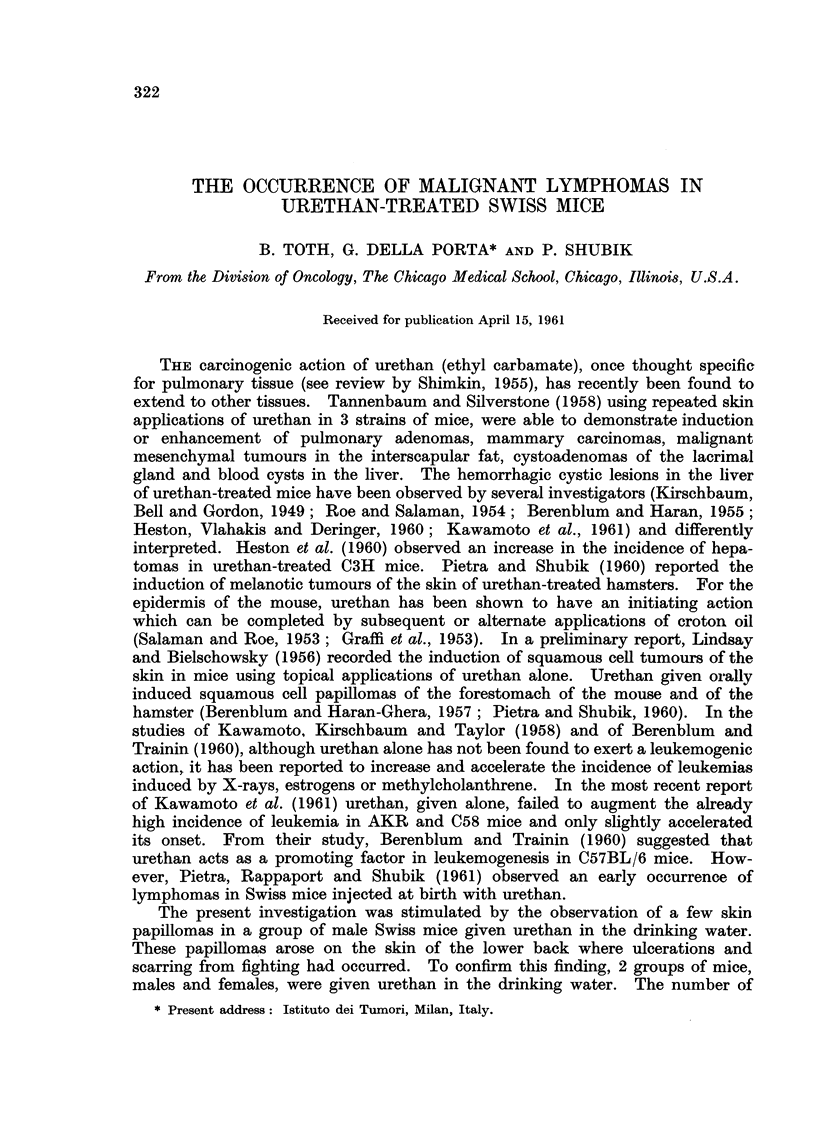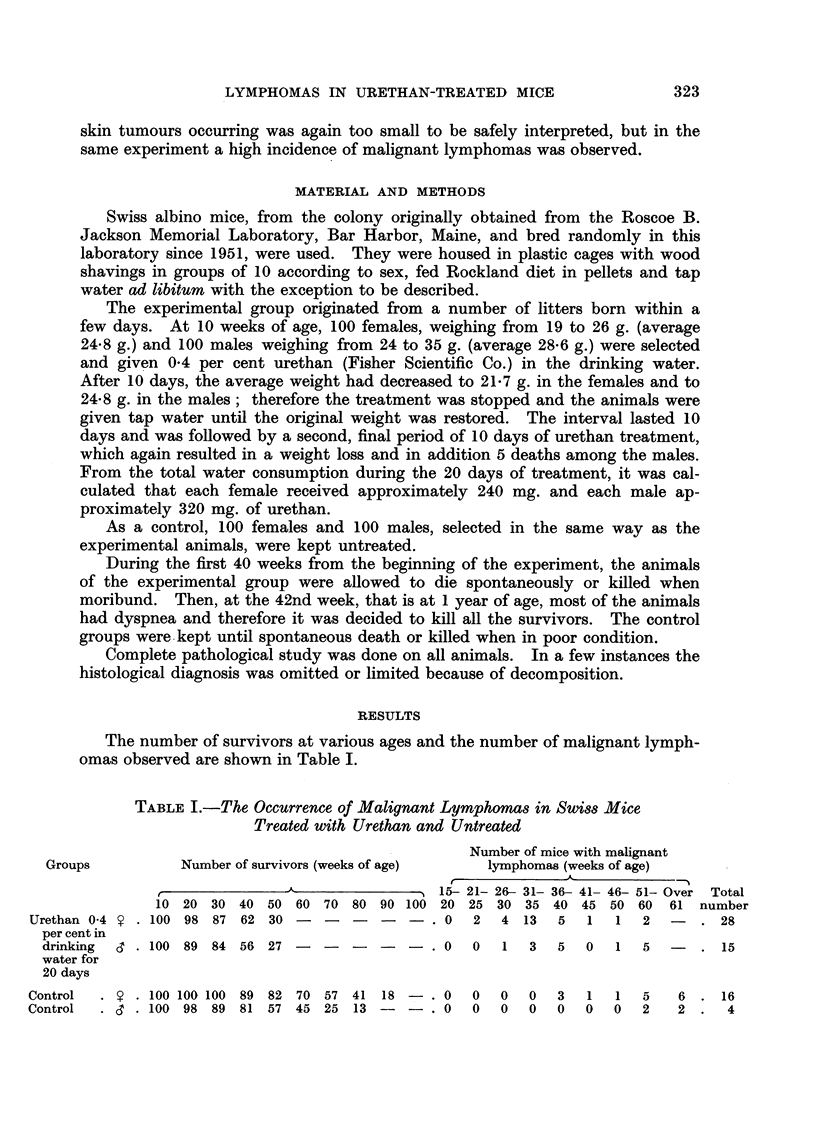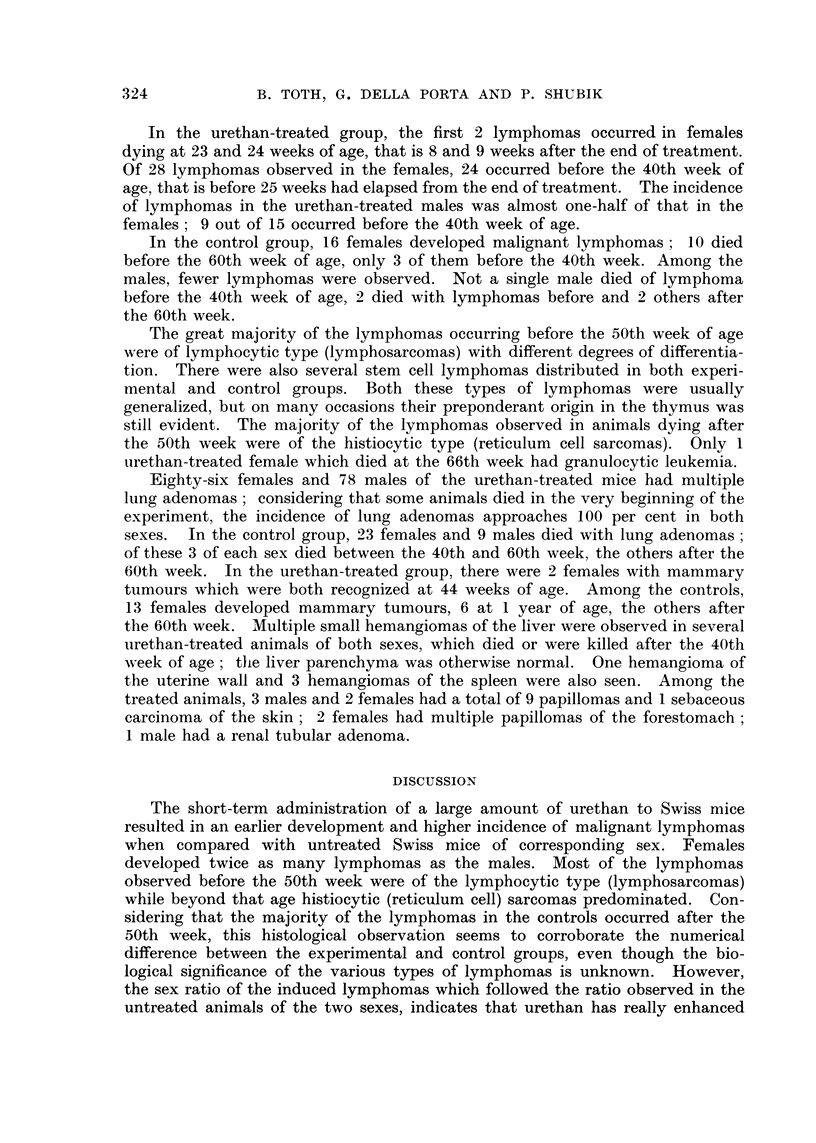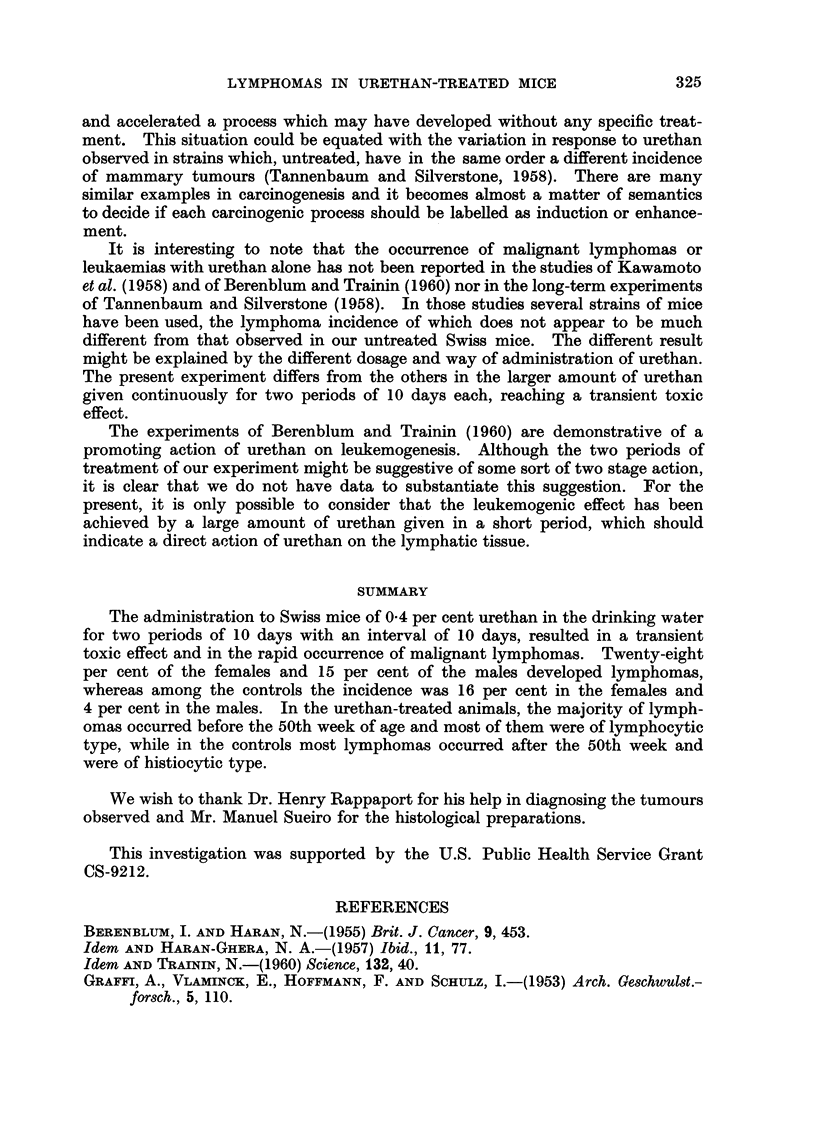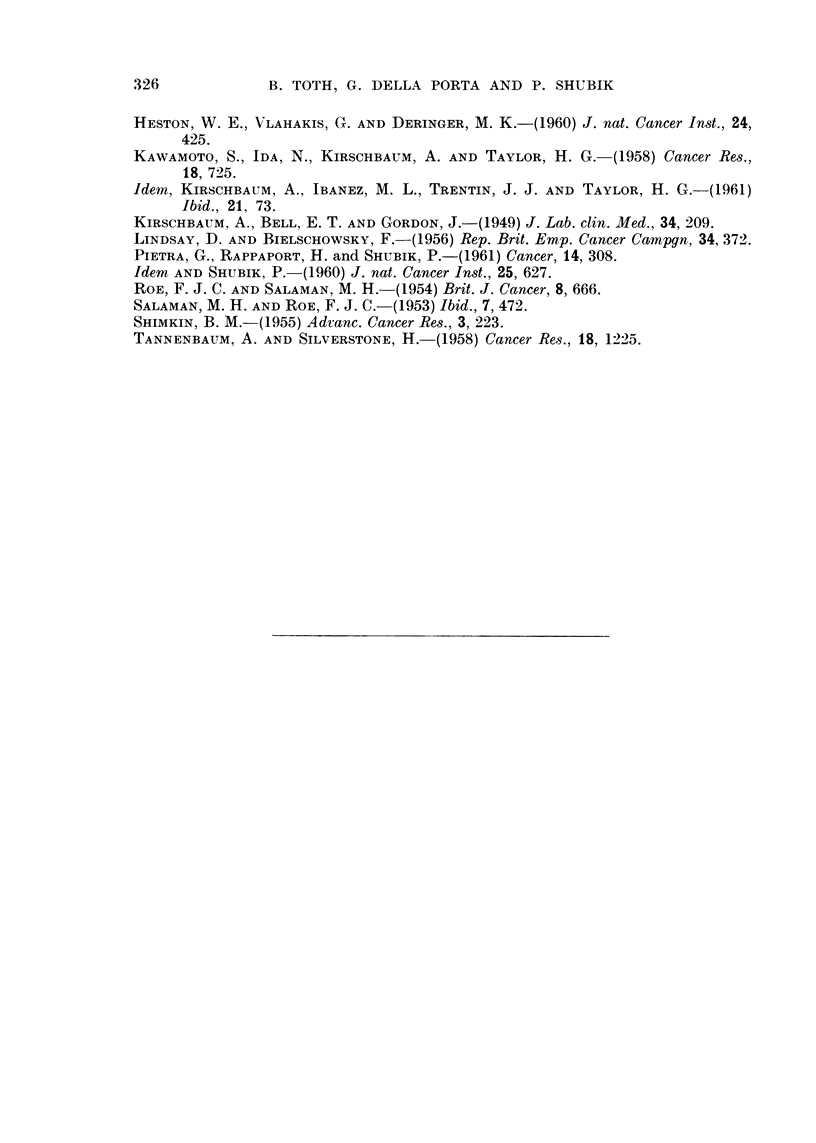# The Occurrence of Malignant Lymphomas in Urethan-treated Swiss Mice

**DOI:** 10.1038/bjc.1961.40

**Published:** 1961-06

**Authors:** B. Toth, G. Della Porta, P. Shubik


### THE OCCURRENCE OF MALIGNANT LYMPHOMAS IN URETHAN-TREATED SWISS MICE

B. TOTH, G. DELLA PORTA* AND P. SHUBIK

*From the Division of Oncology, The Chicago Medical School, Chicago, Illinois, U.S.A.*

Received for publication April 15, 1961

THE carcinogenic action of urethan (ethyl carbamate), once thought specific for pulmonary tissue (see review by Shimkin, 1955), has recently been found to extend to other tissues. Tannenbaum and Silverstone (1958) using repeated skin applications of urethan in 3 strains of mice, were able to demonstrate induction or enhancement of pulmonary adenomas, mammary carcinomas, malignant mesenchymal tumours in the interscapular fat, cystoadenomas of the lacrimal gland and blood cysts in the liver. The hemorrhagic cystic lesions in the liver of urethan-treated mice have been observed by several investigators (Kirschbaum, Bell and Gordon, 1949 ; Roe and Salaman, 1954 ; Berenblum and Haran, 1955 ; Heston, Vlahakis and Deringer, 1960 ; Kawamoto *et al.*, 1961) and differently interpreted. Heston *et al.* (1960) observed an increase in the incidence of hepatomas in urethan-treated C3H mice. Pietra and Shubik (1960) reported the induction of melanotic tumours of the skin of urethan-treated hamsters. For the epidermis of the mouse, urethan has been shown to have an initiating action which can be completed by subsequent or alternate applications of croton oil (Salaman and Roe, 1953 ; Graffi *et al.*, 1953). In a preliminary report, Lindsay and Bielschowsky (1956) recorded the induction of squamous cell tumours of the skin in mice using topical applications of urethan alone. Urethan given orally induced squamous cell papillomas of the forestomach of the mouse and of the hamster (Berenblum and Haran-Ghera, 1957 ; Pietra and Shubik, 1960). In the studies of Kawamoto, Kirschbaum and Taylor (1958) and of Berenblum and Trainin (1960), although urethan alone has not been found to exert a leukemogenic action, it has been reported to increase and accelerate the incidence of leukemias induced by X-rays, estrogens or methylcholanthrene. In the most recent report of Kawamoto *et al.* (1961) urethan, given alone, failed to augment the already high incidence of leukemia in AKR and C58 mice and only slightly accelerated its onset. From their study, Berenblum and Trainin (1960) suggested that urethan acts as a promoting factor in leukemogenesis in C57BL/6 mice. However, Pietra, Rappaport and Shubik (1961) observed an early occurrence of lymphomas in Swiss mice injected at birth with urethan.

The present investigation was stimulated by the observation of a few skin papillomas in a group of male Swiss mice given urethan in the drinking water. These papillomas arose on the skin of the lower back where ulcerations and scarring from fighting had occurred. To confirm this finding, 2 groups of mice, males and females, were given urethan in the drinking water. The number of

* Present address : Istituto dei Tumori, Milan, Italy.

skin tumours occurring was again too small to be safely interpreted, but in the same experiment a high incidence of malignant lymphomas was observed.

### MATERIAL AND METHODS

Swiss albino mice, from the colony originally obtained from the Roscoe B. Jackson Memorial Laboratory, Bar Harbor, Maine, and bred randomly in this laboratory since 1951, were used. They were housed in plastic cages with wood shavings in groups of 10 according to sex, fed Rockland diet in pellets and tap water *ad libitum* with the exception to be described.

The experimental group originated from a number of litters born within a few days. At 10 weeks of age, 100 females, weighing from 19 to 26 g. (average 24·8 g.) and 100 males weighing from 24 to 35 g. (average 28·6 g.) were selected and given 0·4 per cent urethan (Fisher Scientific Co.) in the drinking water. After 10 days, the average weight had decreased to 21·7 g. in the females and to 24·8 g. in the males ; therefore the treatment was stopped and the animals were given tap water until the original weight was restored. The interval lasted 10 days and was followed by a second, final period of 10 days of urethan treatment, which again resulted in a weight loss and in addition 5 deaths among the males. From the total water consumption during the 20 days of treatment, it was calculated that each female received approximately 240 mg. and each male approximately 320 mg. of urethan.

As a control, 100 females and 100 males, selected in the same way as the experimental animals, were kept untreated.

During the first 40 weeks from the beginning of the experiment, the animals of the experimental group were allowed to die spontaneously or killed when moribund. Then, at the 42nd week, that is at 1 year of age, most of the animals had dyspnea and therefore it was decided to kill all the survivors. The control groups were kept until spontaneous death or killed when in poor condition.

Complete pathological study was done on all animals. In a few instances the histological diagnosis was omitted or limited because of decomposition.

### RESULTS

The number of survivors at various ages and the number of malignant lymphomas observed are shown in Table I.

TABLE I.—*The Occurrence of Malignant Lymphomas in Swiss Mice Treated with Urethan and Untreated*

| Groups | | Number of survivors (weeks of age) | | | | | | | | | | Number of mice with malignant lymphomas (weeks of age) | | | | | | | | | |
|---|---|---|---|---|---|---|---|---|---|---|---|---|---|---|---|---|---|---|---|---|---|
| | | 10 | 20 | 30 | 40 | 50 | 60 | 70 | 80 | 90 | 100 | 15–20 | 21–25 | 26–30 | 31–35 | 36–40 | 41–45 | 46–50 | 51–60 | Over 61 | Total number |
| Urethan 0·4 per cent in drinking water for 20 days | ♀ | 100 | 98 | 87 | 62 | 30 | — | — | — | — | — | 0 | 2 | 4 | 13 | 5 | 1 | 1 | 2 | — | 28 |
| | ♂ | 100 | 89 | 84 | 56 | 27 | — | — | — | — | — | 0 | 0 | 1 | 3 | 5 | 0 | 1 | 5 | — | 15 |
| Control | ♀ | 100 | 100 | 100 | 89 | 82 | 70 | 57 | 41 | 18 | — | 0 | 0 | 0 | 0 | 3 | 1 | 1 | 5 | 6 | 16 |
| Control | ♂ | 100 | 98 | 89 | 81 | 57 | 45 | 25 | 13 | — | — | 0 | 0 | 0 | 0 | 0 | 0 | 0 | 2 | 2 | 4 |

In the urethan-treated group, the first 2 lymphomas occurred in females dying at 23 and 24 weeks of age, that is 8 and 9 weeks after the end of treatment. Of 28 lymphomas observed in the females, 24 occurred before the 40th week of age, that is before 25 weeks had elapsed from the end of treatment. The incidence of lymphomas in the urethan-treated males was almost one-half of that in the females ; 9 out of 15 occurred before the 40th week of age.

In the control group, 16 females developed malignant lymphomas ; 10 died before the 60th week of age, only 3 of them before the 40th week. Among the males, fewer lymphomas were observed. Not a single male died of lymphoma before the 40th week of age, 2 died with lymphomas before and 2 others after the 60th week.

The great majority of the lymphomas occurring before the 50th week of age were of lymphocytic type (lymphosarcomas) with different degrees of differentiation. There were also several stem cell lymphomas distributed in both experimental and control groups. Both these types of lymphomas were usually generalized, but on many occasions their preponderant origin in the thymus was still evident. The majority of the lymphomas observed in animals dying after the 50th week were of the histiocytic type (reticulum cell sarcomas). Only 1 urethan-treated female which died at the 66th week had granulocytic leukemia.

Eighty-six females and 78 males of the urethan-treated mice had multiple lung adenomas ; considering that some animals died in the very beginning of the experiment, the incidence of lung adenomas approaches 100 per cent in both sexes. In the control group, 23 females and 9 males died with lung adenomas ; of these 3 of each sex died between the 40th and 60th week, the others after the 60th week. In the urethan-treated group, there were 2 females with mammary tumours which were both recognized at 44 weeks of age. Among the controls, 13 females developed mammary tumours, 6 at 1 year of age, the others after the 60th week. Multiple small hemangiomas of the liver were observed in several urethan-treated animals of both sexes, which died or were killed after the 40th week of age ; the liver parenchyma was otherwise normal. One hemangioma of the uterine wall and 3 hemangiomas of the spleen were also seen. Among the treated animals, 3 males and 2 females had a total of 9 papillomas and 1 sebaceous carcinoma of the skin ; 2 females had multiple papillomas of the forestomach ; 1 male had a renal tubular adenoma.

### DISCUSSION

The short-term administration of a large amount of urethan to Swiss mice resulted in an earlier development and higher incidence of malignant lymphomas when compared with untreated Swiss mice of corresponding sex. Females developed twice as many lymphomas as the males. Most of the lymphomas observed before the 50th week were of the lymphocytic type (lymphosarcomas) while beyond that age histiocytic (reticulum cell) sarcomas predominated. Considering that the majority of the lymphomas in the controls occurred after the 50th week, this histological observation seems to corroborate the numerical difference between the experimental and control groups, even though the biological significance of the various types of lymphomas is unknown. However, the sex ratio of the induced lymphomas which followed the ratio observed in the untreated animals of the two sexes, indicates that urethan has really enhanced and accelerated a process which may have developed without any specific treatment. This situation could be equated with the variation in response to urethan observed in strains which, untreated, have in the same order a different incidence of mammary tumours (Tannenbaum and Silverstone, 1958). There are many similar examples in carcinogenesis and it becomes almost a matter of semantics to decide if each carcinogenic process should be labelled as induction or enhancement.

It is interesting to note that the occurrence of malignant lymphomas or leukaemias with urethan alone has not been reported in the studies of Kawamoto *et al.* (1958) and of Berenblum and Trainin (1960) nor in the long-term experiments of Tannenbaum and Silverstone (1958). In those studies several strains of mice have been used, the lymphoma incidence of which does not appear to be much different from that observed in our untreated Swiss mice. The different result might be explained by the different dosage and way of administration of urethan. The present experiment differs from the others in the larger amount of urethan given continuously for two periods of 10 days each, reaching a transient toxic effect.

The experiments of Berenblum and Trainin (1960) are demonstrative of a promoting action of urethan on leukemogenesis. Although the two periods of treatment of our experiment might be suggestive of some sort of two stage action, it is clear that we do not have data to substantiate this suggestion. For the present, it is only possible to consider that the leukemogenic effect has been achieved by a large amount of urethan given in a short period, which should indicate a direct action of urethan on the lymphatic tissue.

### SUMMARY

The administration to Swiss mice of 0·4 per cent urethan in the drinking water for two periods of 10 days with an interval of 10 days, resulted in a transient toxic effect and in the rapid occurrence of malignant lymphomas. Twenty-eight per cent of the females and 15 per cent of the males developed lymphomas, whereas among the controls the incidence was 16 per cent in the females and 4 per cent in the males. In the urethan-treated animals, the majority of lymphomas occurred before the 50th week of age and most of them were of lymphocytic type, while in the controls most lymphomas occurred after the 50th week and were of histiocytic type.

We wish to thank Dr. Henry Rappaport for his help in diagnosing the tumours observed and Mr. Manuel Sueiro for the histological preparations.

This investigation was supported by the U.S. Public Health Service Grant CS-9212.

### REFERENCES

BERENBLUM, I. AND HARAN, N.—(1955) *Brit. J. Cancer*, **9**, 453.

*Idem* AND HARAN-GHERA, N. A.—(1957) *Ibid.*, **11**, 77.

*Idem* AND TRAININ, N.—(1960) *Science*, **132**, 40.

GRAFFI, A., VLAMINCK, E., HOFFMANN, F. AND SCHULZ, I.—(1953) *Arch. Geschwulst.-forsch.*, **5**, 110.

HESTON, W. E., VLAHAKIS, G. AND DERINGER, M. K.—(1960) *J. nat. Cancer Inst.*, **24**, 425.
KAWAMOTO, S., IDA, N., KIRSCHBAUM, A. AND TAYLOR, H. G.—(1958) *Cancer Res.*, **18**, 725.
*Idem*, KIRSCHBAUM, A., IBANEZ, M. L., TRENTIN, J. J. AND TAYLOR, H. G.—(1961) *Ibid.*, **21**, 73.
KIRSCHBAUM, A., BELL, E. T. AND GORDON, J.—(1949) *J. Lab. clin. Med.*, **34**, 209.
LINDSAY, D. AND BIELSCHOWSKY, F.—(1956) *Rep. Brit. Emp. Cancer Campgn*, **34**, 372.
PIETRA, G., RAPPAPORT, H. and SHUBIK, P.—(1961) *Cancer*, **14**, 308.
*Idem* AND SHUBIK, P.—(1960) *J. nat. Cancer Inst.*, **25**, 627.
ROE, F. J. C. AND SALAMAN, M. H.—(1954) *Brit. J. Cancer*, **8**, 666.
SALAMAN, M. H. AND ROE, F. J. C.—(1953) *Ibid.*, **7**, 472.
SHIMKIN, B. M.—(1955) *Advanc. Cancer Res.*, **3**, 223.
TANNENBAUM, A. AND SILVERSTONE, H.—(1958) *Cancer Res.*, **18**, 1225.